# Umweltgerechtigkeit – Herausforderungen für räumliche Analysen und Monitoring

**DOI:** 10.1007/s00103-025-04118-1

**Published:** 2025-09-01

**Authors:** Gesa Czwikla, Klaus Telkmann, Stefanie Dreger, Gabriele Bolte

**Affiliations:** 1 Institut für Public Health und Pflegeforschung, Abteilung Sozialepidemiologie, Universität Bremen, Grazer Str. 4, 28359 Bremen, Deutschland; 2 Health Sciences Bremen, Universität Bremen, Bremen, Deutschland

**Keywords:** Umweltgerechtigkeit, Umweltexpositionen, Räumliche Epidemiologie, Gesundheit, Monitoring, Environmental justice, Environmental exposures, Spatial epidemiology, Health, Monitoring

## Abstract

Die räumlich-zeitliche Analyse und das Monitoring sozial ungleich verteilter Umweltbelastungen und -ressourcen sowie ihrer gesundheitlichen Wirkungen bilden eine wichtige Grundlage für politische Entscheidungen unter Umweltgerechtigkeitsaspekten. Entsprechende Analyse- bzw. Monitoringansätze sind mit vielfältigen konzeptionellen und methodischen Herausforderungen verbunden, die in diesem Artikel beschrieben und diskutiert werden.

Eine wesentliche Herausforderung liegt in der flächendeckenden Modellierung von Umweltexpositionen, da häufig nur punktuell Messdaten vorliegen und daher komplexe Interpolationsverfahren erforderlich sind. Weitere Herausforderungen bestehen in der Quantifizierung von Ungleichheiten bei Umweltexpositionen, da Umwelt- und Sozialdaten zumeist nur aggregiert auf Ebene administrativer räumlicher Einheiten vorliegen, sowie im Mangel an Daten zur subjektiven Bewertung der Qualität von Umweltexpositionen. Spezifische Herausforderungen eines großräumigen, europaweiten Monitorings gesundheitsrelevanter umweltbezogener Ungleichheiten durch die Weltgesundheitsorganisation (WHO) ergeben sich aus fehlenden Informationen zu relevanten Umweltexpositionen oder Ungleichheitsdimensionen, unterschiedlichen Datenarten sowie fehlenden Quantifizierungen sozialer Unterschiede in der Vulnerabilität.

Die Möglichkeiten, Aussagen über die soziale Verteilung von Umweltexpositionen in der europäischen Region und deren gesundheitliche Wirkungen zu treffen, sind dadurch gegenwärtig begrenzt. Für fundierte politische Entscheidungen und eine evidenzbasierte Reduktion sozial ungleich verteilter Umweltbelastungen und -ressourcen sowie ihrer gesundheitlichen Effekte ist eine deutliche Verbesserung der Datenlage als Voraussetzung für ein umfassendes, einheitliches Monitoring erforderlich.

## Abstract (en)

Spatiotemporal analysis and monitoring of socially unequally distributed environmental hazards and resources as well as their effects on health provide an important basis for political decisions under environmental justice aspects. Appropriate analysis and monitoring approaches are accompanied by diverse conceptual and methodological challenges, which are described and discussed in this paper.

A major challenge lies in the comprehensive modeling of environmental exposures, as measurement data are often only selectively available, requiring complex interpolation methods. Further challenges lie in quantifying inequalities in environmental exposures, as environmental and social data are usually only available in aggregate form at the level of administrative spatial units, and in the lack of data on subjective assessment of the quality of environmental exposures. Specific challenges to large-scale, Europe-wide monitoring of health-relevant environmental inequalities by the World Health Organization (WHO) arise from a lack of information on relevant environmental exposures or inequality dimensions, different types of data, and a lack of quantification of social differences in vulnerability. This currently limits the ability to make statements about the social distribution of environmental exposures in the European Region and their health effects.

In order to make informed political decisions and reduce socially unequally distributed environmental burdens and resources as well as their health effects in an evidence-based manner, a substantial improvement regarding data availability is necessary as a prerequisite for a comprehensive, standardized monitoring.

## Hintergrund

Es gibt umfassende Evidenz, dass sozioökonomisch benachteiligte Bevölkerungsgruppen häufig einer höheren Belastung durch Luftschadstoffe [1–3], Lärm [3, 4] oder Hitze [3, 5–7] ausgesetzt sind und schlechteren Zugang zu gesundheitlichen Ressourcen wie Grün- und Wasserflächen [8] haben. In der Literatur wird die Verbindung dieser höheren Belastung mit unterschiedlichen sozialen Dimensionen aufgezeigt. Dies können z. B. Einkommen, Ethnizität, Geschlecht, Alter, Bildung und Migrationsgeschichte sein. Durch Prozesse der Privilegierung bzw. Benachteiligung sind sozioökonomisch benachteiligte sowie rassistisch diskriminierte Menschen häufiger oder stärker Umweltbelastungen ausgesetzt [9]. Dabei kumulieren oft mehrere Umweltbelastungen, die nicht nur additiv wirken, sondern sich in ihren gesundheitlichen Auswirkungen gegenseitig verstärken. Aus Umweltgerechtigkeitsperspektive werden Umwelt- und Sozialdimensionen daher in ihrer gesundheitlichen Bedeutung gemeinsam betrachtet. Umweltgerechtigkeit ist ein normatives, handlungsorientiertes Leitbild und geht über das reine Beschreiben von sozialen Ungleichheiten bei Umwelt und Gesundheit (*Environmental Health Inequalities*) hinaus [10]. Die meisten Definitionen von Umweltgerechtigkeit beschreiben 3 grundlegende Gerechtigkeitsdimensionen:

- *Verteilungsgerechtigkeit* bezieht sich auf eine gerechte Verteilung von Umweltbelastungen und einen gerechten Zugang zu Umweltressourcen zwischen unterschiedlichen sozialen Gruppen [10, 11].
- *Verfahrensgerechtigkeit* zielt darauf ab, dass alle Menschen gleiche Möglichkeiten haben, an Planungs‑, Anhörungs- und Entscheidungsprozessen zu umweltbezogenen Interventionen zu partizipieren [10, 11].
- *Anerkennungsgerechtigkeit* beruht auf der Erkenntnis, dass die Nichtanerkennung der Lebensweise, der Kultur und der Werte der Menschen, die von Umweltproblemen betroffen sind, Einzelne und die Gemeinschaft abwertet und damit Ungerechtigkeit zulässt [12].

Bei der Erklärung des Zusammenhangs zwischen sozialer Lage, Umweltbelastungen und Gesundheit sind 2 Mechanismen wichtig: *Expositionsvariation* beschreibt die Tatsache, dass verschiedene soziale Gruppen unterschiedlich hohe Expositionen gegenüber Umweltrisiken, wie z. B. Lärm oder Chemikalien, oder unterschiedlich guten Zugang zu Umweltressourcen, wie z. B. Grün- und Erholungsflächen, haben [10]. *Effektmodifikation* bezieht sich auf soziale Unterschiede in der Vulnerabilität hinsichtlich der gesundheitlichen Auswirkungen von Umweltbelastungen. So können Faktoren wie Vorerkrankungen, Zugang zu Informationen, aber auch ökonomische Möglichkeiten, sich individuell gegen Umweltbelastungen zu schützen (z. B. durch den Einbau von Schallschutzfenstern), zu unterschiedlich starken gesundheitlichen Auswirkungen bei gleicher Umweltexposition führen [10]. Soziale Unterschiede in den Fähigkeiten und Verwirklichungschancen (*Capabilities*) spielen hier eine wichtige Rolle [13].

Die Ursprünge der Auseinandersetzung mit Umweltgerechtigkeit (*Environmental Justice*) lassen sich in den USA verorten, in denen Bürgerrechtsaktivist*innen in den frühen 1980er-Jahren dagegen aufbegehrten, dass Mülldeponien und andere umweltbelastende Industrieanlagen überproportional oft in Gegenden angesiedelt wurden, in denen afroamerikanische oder arme Menschen wohnten [14]. In Europa hingegen begann die Auseinandersetzung mit Umweltgerechtigkeitsfragen eher in einem Top-down-Vorgehen, vor allem als Reaktion auf zwischenstaatliche Abkommen, die auf die Förderung und Wahrung der Menschenrechte abzielen [15]. Gleichzeitig rückten in der Forschung zur umweltbezogenen Gesundheit soziale Faktoren stärker in den Fokus [13]. Anders als in den USA hat sich die Umweltgerechtigkeitsdebatte in Europa und speziell in Deutschland nicht primär auf Verhältnisse einer strukturellen rassistischen Benachteiligung und Segregation bezogen, sondern vor allem auf die sozioökonomische Position, charakterisiert anhand von Einkommen und Bildung [15].

Die räumlich-zeitliche Analyse und das Monitoring von sozial ungleich verteilten Umweltbelastungen und -ressourcen sowie deren gesundheitlichen Wirkungen bilden eine wichtige Grundlage für eine normative Bewertung unterschiedlicher Lebensverhältnisse und für politische Entscheidungen zur Schaffung gesunder Lebenswelten für alle [10]. Entsprechend den 3 oben genannten zentralen Umweltgerechtigkeitsdimensionen sollte ein umfassendes Monitoring von Umweltgerechtigkeit kontinuierlich analysieren und bewerten, ob alle Menschen, unabhängig von ihrer sozialen Lage, gleichermaßen:

- vor Umweltbelastungen geschützt sind und Zugang zu Umweltressourcen haben,
- bei umweltrelevanten Planungs‑, Anhörungs- und Entscheidungsprozessen partizipieren können und
- im Bemühen um Umweltgerechtigkeit mitgedacht werden.

Ansätze eines systematischen Monitorings der Dimensionen *Verfahrensgerechtigkeit* und *Anerkennungsgerechtigkeit* sind den Autor*innen dieses Artikels nicht bekannt. Die folgenden Ausführungen beziehen sich daher ausschließlich auf die Dimension *Verteilungsgerechtigkeit.* Für ein kontinuierliches Monitoring von Verteilungsgerechtigkeit kann entweder auf bereits vorhandene Daten zurückgegriffen oder es können neue Daten erhoben werden. Beide Vorgehensweisen sind jeweils mit spezifischen Herausforderungen verbunden.

Das Ziel dieses Diskussionsartikels ist, die Herausforderungen bei der räumlich-zeitlichen Analyse und dem Monitoring von sozial ungleich verteilten Umweltbelastungen und -ressourcen sowie deren gesundheitlichen Wirkungen aufzuzeigen. Ein besonderer Fokus liegt auf den quantitativen Methoden zur Erhebung von Umweltexpositionen, der Erfassung von Expositionsvariation sowie den Herausforderungen eines großräumigen, europaweiten Monitorings gesundheitsrelevanter umweltbezogener Ungleichheiten durch die Weltgesundheitsorganisation (WHO) auf Basis vorhandener Daten.

## Quantitative Methoden zur Erhebung von Umweltexpositionen

Für eine möglichst genaue Erfassung der zu untersuchenden Exposition haben sich im Laufe der Forschung je nach Art der Umweltexposition viele verschiedene Verfahren entwickelt – von rein distanzbasierten Maßen über komplexere Regressionsmodelle bis hin zu modernen Machine-Learning-Algorithmen [16]. Dieser Abschnitt bezieht sich auf objektiv gemessene Umweltexpositionen. Diese werden als räumliche Informationen betrachtet, zumeist in Geoinformationssystemen (GIS) verarbeitet und in Studien zur Abschätzung von umweltbezogenen Gesundheitsrisiken üblicherweise mit den Wohnadressen der Teilnehmer*innen verknüpft.

Eine einfache Methode, das Ausmaß einer Exposition abzuschätzen, bietet die Betrachtung der räumlichen Nähe zur Quelle der Umweltbelastung (z. B. Industrieanlagen [17]) oder die Anzahl der Quellen in einem bestimmten Radius um die Wohnadresse. In ähnlicher Weise lassen sich Satellitendaten nutzen: So beschreiben die Werte des Normalized Difference Vegetation Index (NDVI) den individuellen Grünflächenanteil in der Wohnumgebung. Dabei deuten Werte nahe 1 auf dichte Vegetation hin, während Werte nahe 0 vegetationsfreien Flächen zugeordnet werden und negative Werte Wasserflächen abbilden [18]. Satellitendaten finden zudem auch Anwendung in der Identifizierung von städtischen Hitzeinseln (Urban Heat Islands, UHI) durch großflächige Messung von Oberflächentemperaturen [19].

Lokale Messstationen bieten eine weitere Möglichkeit, Umweltexpositionen zu erheben. Da diese nicht flächendeckend vorhanden sind, nutzt man statistische Verfahren, wie z. B. *Gauß-Prozesse* oder *Kriging,* um Vorhersagen der Expositionslevel an nicht gemessenen Orten zu treffen. Dabei werden die Werte zwischen verschiedenen Messstationen interpoliert. Beispielsweise haben Liang et al. [20] so die Exposition gegenüber Arsen-belastetem Grundwasser für die Lanyang-Ebene im Nordosten Taiwans basierend auf ca. 900 Brunnenstichproben modelliert. In ähnlicher Weise lassen sich Luftschadstoffwerte durch Ausbreitungsrechnung (*Atmospheric Dispersion Modelling*) abschätzen [21].

Regressionsmodelle berücksichtigen weitere Faktoren, die das Ausmaß einer Umweltbelastung beeinflussen können. Insbesondere bei Luftschadstoffen erlaubt *Land Use Regression* (LUR, [22]) eine flächendeckende Zuweisung von Umweltexpositionen basierend auf den gemessenen Werten und zusätzlichen Indikatoren, die die Luftqualität beeinflussen. So wurden in 20 europäischen Studienregionen im Rahmen des ESCAPE-Projekts (European Study of Cohorts for Air Pollution Effects)^1^ Werte für Feinstaubbelastung (PM_2,5_, PM_10_) mittels LUR für die Wohnadressen der Studienteilnehmer*innen modelliert. Als Datengrundlage dienten hier gemessene Feinstaubkonzentrationen an 20 bis 40 Messstationen pro Studienregion sowie Informationen zu Verkehrsaufkommen, Bevölkerungsdichte, Flächennutzung und Geländeprofilen [23]. Dieses generelle Vorgehen lässt sich auch zur Modellierung von Lärm [24] oder Hitzebelastung nutzen. Ketterer und Matzarakis [25] haben damit beispielsweise hochauflösende Karten für die UHI-Intensität und die physiologisch äquivalente Temperatur in Stuttgart berechnet.

Eine mögliche Limitierung dieser Art der Modellierung von räumlichen Daten wird durch „Tobler’s first law of geography“ verdeutlicht: „Everything is related to everything else, but near things are more related than distant things“ [26]. In der räumlichen Statistik ist diese Eigenschaft bekannt als *räumliche Autokorrelation*. Das heißt, dass Expositionswerte in einer Region einen Einfluss auf die Expositionswerte für umliegende Regionen haben können. Die für Regressionsmodelle benötigte Annahme der Unabhängigkeit der Beobachtungen ist also womöglich verletzt. Zudem kann eine räumliche Heterogenität zur Folge haben, dass konstante Regressionskoeffizienten ihre Validität verlieren [27]. Um sowohl die räumliche als auch die zeitliche Variation in solchen räumlichen Regressionsmodellen explizit zu berücksichtigen, lassen sich komplexere Verfahren wie *Geographically Weighted Regression* [28] verwenden. Shen et al. [29] z. B. haben ein europaweites Luftschadstoffmodell berechnet, das sowohl die zeitliche Änderung (2000 bis 2019) sowie mögliche geografische Unterschiede berücksichtigt. Die Autor*innen führen als Beispiel an, dass das Verkehrsaufkommen unterschiedliche Assoziationen mit Stickstoffdioxid-(NO_2_-)Werten in Süd- und Nordeuropa aufweisen kann.

Die beschriebenen Methoden zur Expositionserfassung sind statisch und berücksichtigen weder Mobilität noch Aufenthaltsdauer einzelner Personen. Für eine umfassende Beschreibung der persönlichen Umweltexposition müssten diese Faktoren durch *Zeit-Aktivitäts-Muster* miterfasst werden. Für eine individuelle Messung von Luftschadstoffbelastungen werden Studienteilnehmer*innen beispielsweise mit einem tragbaren Messgerät ausgestattet, das diese Daten in Echtzeit erfasst. Jedoch sind die Stichprobengrößen bei solchen Untersuchungen üblicherweise eher gering. Eine mit weniger Aufwand verbundene Möglichkeit bietet die Aufzeichnung von Global-Positioning-System-(GPS-)Daten durch Smartphones, um Aufenthaltsort und -dauer der Teilnehmer*innen zu erfassen und mit Daten zu Luftschadstoffen zu verknüpfen [30]. Auch die persönliche Hitzebelastung lässt sich nicht nur durch die Temperatur an der Wohnadresse beschreiben, sondern hängt von weiteren Faktoren wie der Isolierung der Wohnung und dem individuellen Verhalten der Bewohner*innen ab, die einen Einfluss auf das tatsächliche Ausmaß und die Dauer der Exposition gegenüber Hitze haben [31].

## Herausforderungen bei der Quantifizierung von sozialen Ungleichheiten bei Umweltexpositionen

Die einfachste Möglichkeit, soziale Ungleichheiten bei Umweltexpositionen zu untersuchen, ist es, die geografischen Verteilungen von einzelnen Variablen miteinander zu vergleichen und deren Assoziationen zu berechnen. Maantay und Maroko [32] haben so untersucht, ob Ethnizität eine Rolle in Bezug auf die Exposition gegenüber Überschwemmungen in New York spielt.

Ein wesentlicher Aspekt ist hier die räumliche Auflösung bei aggregierten Daten auf kontextueller Ebene. Informationen zu Umweltexpositionen, Bevölkerungsdichte, Arbeitslosenquote, Medianeinkommen oder Flächennutzung liegen zumeist auf Ebene administrativer räumlicher Einheiten vor, wie beispielsweise Baublock, Ortsteil, Gemeinde oder Landkreis. In natürlicher Weise unterscheiden sich die Werte der aggregierten Daten je nach Granularität, was im Allgemeinen als *Modifiable Areal Unit Problem* (MAUP) bekannt ist. Aggregierte Maße bezogen auf administrative Einheiten suggerieren jeweils eine gewisse Homogenität der untersuchten Variablen innerhalb jeder Einheit. Falls die räumliche Auflösung zu grob ist, kann es sein, dass diese Werte sich in verschiedenen Teilbereichen einer Einheit stark unterscheiden. So können sich beispielsweise der Grünflächenanteil oder auch sozioökonomische Indikatoren in einigen Teilbereichen stark vom Mittelwert der untersuchten administrativen Einheit unterscheiden. Dies kann zu einer Verzerrung der Ergebnisse und bei der Verknüpfung mit individuellen Daten zu einem ökologischen Fehlschluss führen [33]. Es ist wünschenswert, möglichst kleinräumige Daten vorliegen zu haben. Jedoch sind diese häufig nicht verfügbar. Parenteau und Sawada [34] haben am Beispiel von 3 unterschiedlich gewählten administrativen räumlichen Einheiten gezeigt, dass Assoziationen von NO_2_-Belastung und Atemwegserkrankungsraten in Kanada maßgeblich von der Wahl der räumlichen Auflösung abhängig sind. In dem vom Umweltbundesamt beauftragten Gutachten zu sozialen Unterschieden in der Feinstaubexposition in Deutschland weisen Gaffron und Freude [35] auf die Problematik hin, dass Daten für den inhaltlich gut geeigneten Indikator Haushaltseinkommen nur grob aufgelöst (1 × 1 km^2^) verfügbar sind, wohingegen Daten für den Miet- und Kaufspiegel zwar eine für die Analyse kleinräumiger Unterschiede geeignete Auflösung haben (Baublockebene), jedoch inhaltlich weniger Aussagekraft für die Charakterisierung des sozioökonomischen Status der Bevölkerung bieten.

Wenn Umweltdaten in einer räumlichen Auflösung bezogen auf durch administrative Grenzen definierte Räume vorliegen, stellt sich die Frage, ob diese Räume (*Space *als traditionelle euklidische Konzeption) den Lebensrealitäten der Menschen (*Place* aus relationaler Sicht) entsprechen [36]. Corburn [37] empfiehlt daher für Analysen zu gesundheitlicher Chancengerechtigkeit im urbanen Raum die komplexen Interaktionen zwischen physischen Merkmalen eines Raumes und dessen Wahrnehmung und Gestaltung durch die Bewohner*innen und die Politik zu berücksichtigen.

Eine zusätzliche Schwierigkeit bei der statistischen Analyse von Umwelteffekten ist die bereits angesprochene räumliche Autokorrelation. So weisen möglicherweise auch gesundheitsbezogene Zielgrößen wie Mortalität diese Muster auf [38]. Nutzt man Regressionsmodelle, um Vorhersagen zu Gesundheitsrisiken durch Umweltexpositionen adjustiert für andere Risikofaktoren zu treffen, sollte dies berücksichtigt werden. Falls die Verteilung der Residuen räumliche Autokorrelation aufweist, kann die Anwendung spezieller räumlicher Regressionsmodelle zuverlässigere Schätzungen liefern [39].

Eine weitere Herausforderung ist, dass manche Daten zur Umweltsituation keine Bewertung hinsichtlich deren Qualität zulassen. Insbesondere bei Grünflächen kann dies zu Problemen bei der Interpretation der Ergebnisse führen. So messen NDVI-Werte zwar objektiv den Anteil an Grün in der Umgebung, lassen jedoch ohne weitere Daten keine Rückschlüsse darauf zu, ob es sich um für alle öffentlich zugängliche Grünflächen handelt und diese von ausreichender Qualität sind. Gerade hier sind jedoch Qualität und Nutzbarkeit ein entscheidender Faktor für gesundheitsrelevante Effekte [40].

Für die Entscheidungsunterstützung im Kontext von Umweltgerechtigkeit als handlungsorientiertes Leitbild ist es essenziell, die Gesamtsituation in einem Lebensraum (*Place*) beurteilen zu können. Hierfür sind Informationen zum Ausmaß der sozialen Ungleichheiten bestenfalls von allen gesundheitlich relevanten Umweltfaktoren und deren Interaktionen im Sinne eines Exposom-Konzeptes notwendig [41, 42] wie auch Abschätzungen der sozialen Ungleichheiten in der Vulnerabilität der Wohnbevölkerung, die sich im Sinne der Effektmodifikation als Unterschiede in den gesundheitlichen Wirkungen einer bestimmten Expositionskonstellation manifestieren. Die Forschung steht hierzu noch relativ am Anfang. Im Bereich des Monitorings mit vorhandenen Daten zu Umweltbelastungen bzw. -ressourcen und zur sozialen Lage der Bevölkerung in einem (urbanen) Raum werden verschiedene Indizes eingesetzt, sei es durch Kombination mehrerer Umweltaspekte, mehrerer Sozialdimensionen (Deprivationsindizes) oder von Umwelt- und Sozialdaten. Insbesondere auf kommunaler Ebene wird ein kleinräumiges, integriertes Monitoring als wesentlicher Baustein für eine informierte Entscheidungsfindung eingeschätzt [43]. Die aktuellen Entwicklungen und Diskussionen zur Aussagekraft von Indizes und verschiedenen Ansätzen eines kleinräumigen integrierten Monitorings sind nicht Gegenstand dieses Beitrags.

## Herausforderungen beim Monitoring von Umweltgerechtigkeit mit vorhandenen Daten

Im Folgenden wird der Monitoringansatz der Weltgesundheitsorganisation (WHO) zu gesundheitsrelevanten umweltbezogenen Ungleichheiten in Europa, der auf vorhandenen Daten basiert, vorgestellt und seine zentralen Herausforderungen diskutiert.

### Monitoringansatz der WHO zu gesundheitsrelevanten umweltbezogenen Ungleichheiten in Europa

Der Grundstein für das Monitoring wurde mit 2 in den Jahren 2012 [44] und 2019 [45] von der WHO/Europa veröffentlichten Sachstandsberichten gelegt. Eine Fortsetzung findet seit dem Jahr 2022 mit Faktenblättern statt, die in regelmäßigen Abständen vom WHO Collaborating Centre for Environmental Health Inequalities am Institut für Public Health und Pflegeforschung der Universität Bremen erarbeitet werden.^2^ Ziel des Monitorings ist, das Ausmaß gesundheitsrelevanter umweltbezogener Ungleichheiten innerhalb der 53 Mitgliedstaaten der europäischen Region der WHO zu quantifizieren, Trends bei sozialen Ungleichheiten in Umweltexpositionen oder umweltbezogenen Gesundheitsoutcomes abzuschätzen und die am stärksten von Ungleichheiten betroffenen Bevölkerungsgruppen zu identifizieren. Dadurch soll eine Evidenzgrundlage für Maßnahmen auf nationaler oder lokaler Ebene geschaffen und den Mitgliedsstaaten eine informierte Entscheidungsfindung ermöglicht werden.

Das Monitoring des Sachstandsberichts der WHO/Europa aus dem Jahr 2019 [45] besteht aus 18 Indikatoren in den Bereichen *städtische Umwelt und Transport, Wohnbedingungen, Grundversorgung, Verletzungen* und *Arbeitsbedingungen* sowie einem Indikator zur Exposition gegenüber chemischen Substanzen. Darauf aufbauend wurden bislang (Stand: 05/2025) 9 Faktenblätter veröffentlicht.^3^ Das Monitoring erfolgt auf Grundlage harmonisierter Daten aus internationalen Datenbanken. Diese haben den Vorteil, dass sie relativ schnell zugänglich sind und überwiegend kostenfrei zur Verfügung stehen. Herausforderungen bestehen insbesondere im Umgang mit *fehlenden Informationen zu relevanten Umweltexpositionen oder Ungleichheitsdimensionen, unterschiedlichen Datenarten* sowie *fehlenden Quantifizierungen sozialer Unterschiede in der Vulnerabilität*.

### Fehlende Informationen zu relevanten Umweltexpositionen

Die europäische Region der WHO erstreckt sich geografisch von Grönland bis zur Russischen Föderation und vom Mittelmeer bis zur Ostsee [46]. Für einige der 53 Mitgliedsstaaten, insbesondere jene im östlichen Teil der Region, liegen in internationalen Datenbanken keine Daten vor, da in diesen Ländern häufig keine harmonisierten, internationalen Erhebungen durchgeführt werden. Die „EU Statistics on Income and Living Conditions (EU-SILC)“ zum Beispiel und die „Airbase – European Air Quality Database“ der Europäischen Umweltagentur (EEA) beinhalten Daten zu lediglich etwas mehr als der Hälfte aller Mitgliedsstaaten: Während für alle 27 Mitgliedsländer der Europäischen Union (EU) Daten vorliegen, zeigen sich vor allem Datenlücken für einen Teil der Nicht-EU-Länder West- und Südosteuropas. Daten für Länder der Gemeinschaft Unabhängiger Staaten (inkl. Georgien und Ukraine) liegen in der Datenbank gar nicht vor. Globale Datenbanken der WHO oder der Vereinten Nationen, wie beispielsweise die „WHO Ambient Air Quality Database“, bieten den Vorteil, dass sie Informationen für einen Großteil der 53 Mitgliedsstaaten beinhalten. Sie weisen jedoch einige methodische Limitationen auf, die ihre Eignung für ein Monitoring infrage stellen. So basieren die Luftschadstoffjahresmittelwerte für einige Länder lediglich auf Messwerten weniger Städte oder Monate. Zudem zeigt sich eine große Heterogenität bei den angewandten Messmethoden sowie in der Qualität der Messungen. Ein valider direkter Vergleich der Länder ist dadurch nicht immer möglich [47].

### Fehlende Informationen zu relevanten Ungleichheitsdimensionen

Gesundheitliche Ungleichheiten lassen sich anhand verschiedener Dimensionen (z. B. Einkommen, Armutsbetroffenheit, Geschlecht, Bildung, Haushaltszusammensetzung, Wohnort) erfassen. Bei Analysen mit vorhandenen Daten hängt die Wahl geeigneter Ungleichheitsdimensionen nicht nur von inhaltlichen Überlegungen, sondern vor allem von Fragen der Datenverfügbarkeit ab. In dem bereits genannten EU-SILC-Datensatz liegen Umweltdaten aufgeschlüsselt für die Mehrheit der zuvor genannten Ungleichheitsdimensionen vor. In Datenbanken der Umweltüberwachung, wie der „Airbase – European Air Quality Database“ der EEA, liegen jedoch Informationen zu Ungleichheitsaspekten nicht bzw. nur eingeschränkt vor. Möglichkeiten zur Stratifizierung bleiben hier auf den Wohnort begrenzt (lediglich Vergleich städtisch/ländlich möglich). Die EEA hat daher Luftschadstoffdaten mit Daten zum Bruttoinlandsprodukt (BIP), die in der „Eurostat“-Datenbank enthalten sind, verknüpft [48]. Daten zur Feinstaubbelastung liegen in einer räumlichen Auflösung von 1 × 1 km^2^ vor. Da keine europaweiten Daten zum BIP auf einer kleineren Ebene als NUTS‑3^4^ vorliegen, ist NUTS‑3 die kleinste räumliche Einheit, auf der eine Verknüpfung der beiden Datenquellen möglich ist. In ihrem Analyseansatz vergleicht die EEA die Exposition gegenüber PM_2,5_ zwischen den 20 % der NUTS-3-Regionen in der EU mit dem niedrigsten bzw. höchsten BIP. Es wird berichtet, dass PM_2,5_-Konzentrationen in den Regionen mit dem niedrigsten BIP um etwa ein Drittel höher sind, und daraus geschlussfolgert, dass Ungleichheiten zwischen ärmeren und reicheren Regionen in der EU bestehen [48].

Für das Monitoring der WHO wurde der Ansatz der EEA auf die einzelnen Länder der EU angewandt. Hier zeigte sich ein anderes Bild: In den meisten Ländern waren Bevölkerungsgruppen, die in den 20 % der Regionen mit dem höchsten BIP leben, stärker gegenüber PM_2,5_ exponiert. Für NO_2_ waren diese Unterschiede noch deutlicher ausgeprägt. Aus dieser Beobachtung könnte man schlussfolgern, dass nicht ärmere, sondern reichere Bevölkerungsgruppen in größerem Umfang gesundheitsschädlichen Luftschadstoffen ausgesetzt sind. Der hier beschriebene Ansatz ist zur Analyse sozioökonomischer Ungleichheiten in der Luftschadstoffbelastung aus Sicht der Autor*innen dieses Beitrags jedoch in mehrerlei Hinsicht problematisch. Zum einen greift das weiter oben angesprochene MAUP, da davon auszugehen ist, dass die Belastung durch Luftschadstoffe je nach lokalen Gegebenheiten (z. B. Nähe zu Hauptverkehrsstraßen) innerhalb von NUTS-3-Regionen variiert. Zum anderen sagt die Höhe des BIP einer NUTS-3-Region zwar etwas über deren wirtschaftliche Leistungsfähigkeit, jedoch wenig über die sozioökonomische Lage ihrer Wohnbevölkerung aus. So ist beispielsweise ein hohes BIP einer NUTS-3-Region nicht mit einem auskömmlichen Einkommen oder vorhandenem Vermögen der in der Region lebenden Bevölkerung gleichzusetzen. Um Aussagen über sozioökonomische Ungleichheiten in der Luftschadstoffbelastung treffen zu können, bedarf es besser geeigneter Indikatoren zur Beschreibung der sozioökonomischen Lage verschiedener Bevölkerungsgruppen und Expositionsdaten in höherer räumlicher Auflösung, die auf europäischer Ebene jedoch aktuell nicht verfügbar sind.

### Unterschiedliche Datenarten

Der Monitoringansatz der WHO basiert auf unterschiedlichen Datenarten, darunter von Behörden veröffentlichten Umweltdaten, wie z. B. Daten zur Luftschadstoffbelastung der EEA, aber auch Befragungsdaten, wie z. B. Daten der europäischen Gesundheitsumfrage (European health interview survey EHIS), die für alle Mitgliedsländer der EU verpflichtend ist. Ein häufiges Problem bei Befragungsdaten ist die sogenannte Non-Response. Es besteht die Gefahr einer Verzerrung, wenn sich an einer Befragung teilnehmende Personen von nichtteilnehmenden Personen systematisch unterscheiden und infolgedessen die Validität der Ergebnisse eingeschränkt ist [50]. Darüber hinaus können sich die Datenarten auch innerhalb einer Datenquelle zwischen den Ländern unterscheiden. In der EU-SILC beispielsweise sind für einige Länder ausschließlich Befragungsdaten, für andere Länder auch administrative Daten bzw. Registerdaten enthalten [51]. Ein kontinuierliches Monitoring wird zudem erschwert, wenn relevante Indikatoren auf Daten von Befragungen, die nur in größeren zeitlichen Abständen durchgeführt werden, oder auf sogenannten Ad-hoc-Modulen, die nicht Teil des Standardfragenprogramms einer Befragung sind, basieren. Fehlt es an aktuellen Daten, steht dies dem Ziel entgegen, eine Evidenzgrundlage für Maßnahmen auf nationaler oder lokaler Ebene zu schaffen und den Mitgliedsstaaten eine informierte Entscheidungsfindung zu ermöglichen.

### Fehlende Quantifizierungen sozialer Unterschiede in der Vulnerabilität

Der Monitoringansatz der WHO umfasst derzeit in erster Linie Expositionsunterschiede. Das Global Health Observatory der WHO^5^ stellt allerdings auch Daten zur globalen Krankheitslast aufgrund von Luftverschmutzung zumindest stratifiziert nach Geschlecht bereit. Diese werden mittels *vergleichender Risikoschätzung* (engl. Comparative Risk Assessment; [52]) anhand von Daten zur Luftschadstoffexposition sowie relativen Risiken für den Zusammenhang zwischen Luftschadstoffbelastung und relevanten Gesundheitsoutcomes, die epidemiologischen Studien entnommen werden, ermittelt. Insgesamt sind aufgrund fehlender Daten zu Effektschätzern für verschiedene soziale Gruppen bzw. verschiedene Konstellationen sozialer Unterschiede in der Vulnerabilität die Möglichkeiten, die gesundheitlichen Effekte von Expositionen im Zusammenwirken mit Vulnerabilitätsunterschieden abzuschätzen, begrenzt. Für eine solche umfassende Abschätzung besteht ein Bedarf an epidemiologischen Studien, in denen hinsichtlich Effektmodifikation zwischen relevanten sozialen Gruppen ausgewertet und berichtet wird.

## Fazit

Die Analyse von sozial ungleich verteilten Umweltexpositionen und deren gesundheitlichen Effekten ist eine wesentliche Grundlage für eine normative Bewertung unterschiedlicher Lebensverhältnisse sowie für politische Entscheidungen, die darauf abzielen, gesunde Lebenswelten für alle zu schaffen. Dieser Beitrag zeigt vielfältige konzeptionelle und methodische Herausforderungen bei der Analyse und dem Monitoring von sozialen Ungleichheiten bei Umweltexpositionen und deren gesundheitlicher Bedeutung im Kontext von Umweltgerechtigkeit auf.

Bezogen auf den Monitoringansatz der WHO sind gegenwärtig die Möglichkeiten, für die europäische Region der WHO Aussagen über die soziale Verteilung von Umweltexpositionen sowie über deren gesundheitliche Effekte im Zusammenwirken mit Vulnerabilitätsunterschieden zu treffen, begrenzt. Verbesserungsbedarf besteht insbesondere bei der Verfügbarkeit aktueller, international vergleichbarer Expositionsdaten, die für möglichst viele relevante Ungleichheitsdimensionen aufgeschlüsselt und in geeigneter räumlicher Auflösung vorliegen. Ebenso fehlen epidemiologische Studien, in denen Effektmodifikationen zwischen relevanten sozialen Gruppen ausgewertet und berichtet werden. Ein Teil der im Zusammenhang mit dem Monitoringansatz der WHO genannten Limitationen liegt vor allem darin begründet, dass für die gesamte Region nicht genügend vergleichbare Daten vorhanden sind.

Der Umweltgerechtigkeitsansatz beinhaltet eine normative Bewertung beobachteter Ungleichverteilungen, auf deren Grundlage Handlungsbedarfe und politische Entscheidungen abzuleiten sind. Hierbei ist zu berücksichtigen, dass beobachtete Ungleichheiten, wie z. B. in Städten zuweilen beobachtete höhere Luftschadstoff- und Lärmbelastungen an der Wohnadresse von sozial Bessergestellten, nicht per se ungerecht sein müssen. Dies ist beispielsweise dann der Fall, wenn entsprechende Bevölkerungsgruppen die ökonomischen Möglichkeiten und Handlungsspielräume haben, ihre Expositionen gegenüber Luftschadstoffen und Lärm individuell zu reduzieren (z. B. Schlafzimmer zur straßenabgewandten Seite, Lärmschutzfenster, Umzug, Zweitwohnsitz).

Aus einer Umweltgerechtigkeitsperspektive bedarf es der Ergänzung und Qualifizierung epidemiologischer Expositionsforschung und quantitativer Monitoringansätze durch die Erfahrungen und Wissensbestände der Menschen vor Ort. Van Horne et al. [53] schlagen ein „environmental justice framework for exposure science“ als neuen Standard vor. Dies beinhaltet einen gemeinsamen Forschungsprozess von Menschen aus einem Stadtteil oder einer Region, verschiedenen Akteur*innen, Interessenvertreter*innen und Wissenschaftler*innen. So kann es gelingen, die strukturellen Prozesse, die zu Mehrfachbelastungen und Ungerechtigkeiten führen, umfassend zu verstehen und zu politischem Handeln für mehr Umweltgerechtigkeit beizutragen.

## Footnotes

1 http://www.escapeproject.eu/.

2 https://www.uni-bremen.de/who-collaborating-centre-for-environmental-health-inequalities. Zugegriffen am 01.08.2025.

3 https://www.uni-bremen.de/who-collaborating-centre-for-environmental-health-inequalities/faktenblaetter. Zugegriffen am 01.08.2025.

4 NUTS‑3 ist eine von 3 Hierarchiestufen der *Klassifikation der Gebietseinheiten für die Statistik* (Nomenclature des Unités territoriales statistiques, NUTS) und entspricht mit einer Bevölkerungszahl zwischen 150.000 und 800.000 Einwohner*innen in Deutschland den Kreisen und kreisfreien Städten [49].

5 https://www.who.int/data/gho/gho.
